# In Vitro Digestion of Peanut Skin Releases Bioactive Compounds and Increases Cancer Cell Toxicity

**DOI:** 10.3390/antiox12071356

**Published:** 2023-06-28

**Authors:** Karina Cordeiro-Massironi, Rosana Aparecida M. Soares-Freitas, Geni Rodrigues Sampaio, Ana Clara da C. Pinaffi-Langley, Raquel Bridi, Adriano Costa de Camargo, Elizabeth Aparecida F. S. Torres

**Affiliations:** 1 Department of Nutrition, School of Public Health, University of São Paulo, São Paulo 01246-904, Brazil; kcmassironi@gmail.com (K.C.-M.); rosanaso@usp.br (R.A.M.S.-F.); genirs@usp.br (G.R.S.); 2 Department of Nutrition Sciences, College of Allied Health, University of Oklahoma Health Sciences Center, Oklahoma City, OK 73117, USA; napinaffi@gmail.com; 3 Departamento de Química Farmacológica y Toxicológica, Facultad de Ciencias Químicas y Farmacéuticas, Universidad de Chile, Santiago 8380000, Chile; raquelbridi@ciq.uchile.cl; 4 Nutrition and Food Technology Institute, University of Chile, Santiago 7830490, Chile

**Keywords:** *Arachis hypogaea*, polyphenols, diabetes mellitus, obesity, phytochemicals, cell survival, HCT116 cells

## Abstract

Peanut skin is a rich source of bioactive compounds which may be able to reduce the risk factors associated with metabolic syndromes. This study aimed to characterize bio-compounds from peanut skin (*Arachis hypogaea*) and their bioactivity (antioxidant activity, inhibition of lipase, and carbohydrase enzymes) and to evaluate their anti-proliferative properties in colorectal cancer cells (HCT116) upon in vitro digestion. Peanut skin was digested in two sequential phases, and the final content, named phase-1 (P1) and phase-2 (P2) extracts, was evaluated. Several bioactive compounds were positively identified and quantified by liquid chromatography, including quinic acid, released especially after in vitro digestion. The total phenolic content and, regardless of the method, the antioxidant activity of P1 was higher than P2. P1 also showed a lower enzyme inhibitory concentration IC_50_ than P2, lipase, and α-glucosidase. For cell viability in HCT116 cells, lower concentrations of P1 were found for IC_50_ compared to P2. In conclusion, bioactive compounds were released mainly during the first phase of the in vitro digestion. The digested samples presented antioxidant activity, enzyme inhibitory activity, and cancer cell cytotoxicity, especially those from the P1 extract. The potential applications of such a by-product in human health are reported.

## 1. Introduction

Nuts and oilseeds, such as peanuts and almonds, are considered part of a healthy diet that has been associated with a reduced risk of developing chronic noncommunicable diseases [1]. Several health benefits are associated with the intake of these foods, such as the improvement of important cardiovascular disease biomarkers [2,3], as well as inverse relationships between nut and oilseed intake and cardiovascular, respiratory, and cancer mortality [2,4].

Peanuts (*Arachis hypogaea*) are a legume crop considered an oilseed due to their high-fat content. Its nutrient composition includes a high content of unsaturated fatty acids, protein, fiber, and bioactive compounds, such as phenolics and tocopherols. Among phenolic compounds, peanuts contain protocatechuic, p-coumaric, caffeic, quinic, ferulic, sinapic, coutaric, and ellagic acids; they are quercetin, resveratrol, catechin, epicatechin, and proanthocyanidins free, and are esterified, etherified and/or insoluble-bound [5,6].

Peanuts are ubiquitously consumed in several different forms and preparations. According to data from Atlasbig 2018–2022, the world produces approximately 48 million tons of peanuts yearly, with China as the biggest producer. Brazil produces around 580 thousand tons of peanuts per year, with most as the Runner IAC-886 cultivar from the Virginia group, which contains high levels of oleic acid [7]. According to a national population survey POF 2017–2018, Brazilians consume, on average, 1.154 kg/year of nuts and oilseeds (including peanuts) per capita [8].

The industrial processing of peanuts generates by-products comprising mainly skins and shells, which are underutilized for human consumption. Instead, a small fraction of this residue is utilized for the extraction of phenolic compounds and nutraceuticals or in animal feed. However, there has been a recent change in focus to improve the usage of industrial by-products, including those of peanut processing [9].

The health effects of peanut consumption are usually associated with the antioxidant properties of its phenolic compounds [6]. Previous studies have reported that peanuts present anti-obesity and anti-diabetic properties [10,11], protective properties against Alzheimer’s disease, and anticancer activity [9], especially against colon cancer [12].

Peanuts are also an excellent source of proanthocyanidins and catechins, in addition to such compounds as resveratrol, which both act as antioxidants that may protect against heart disease, inflammation, and cardiovascular diseases, such as arteriosclerosis, in addition to being a chemopreventive agent in some types of cancer. When roasted and if consumed with the skin, the antioxidant capacity of peanuts is doubled [9,13].

Regarding metabolic diseases, such as diabetes and obesity, peanuts may inhibit digestive enzymes (e.g., lipase and α-glucosidase), representing an efficient strategy to lower post-prandial hyperglycemia and reduce dietary lipid absorption. Moreover, this strategy may contribute to weight management, reduction in adipose tissue accumulation, and lowering of oxidative stress related to diabetes, obesity, and other risk factors associated with metabolic syndromes [11].

The study of the bioactive effects of foods must include the development of a mechanism to understand the influence of the food matrix and composition on human health, for instance, by simulating the digestion process in the upper gastrointestinal tract. Although several studies have investigated the biological potential of phenolic-rich peanut skin extracts [14,15,16], to our knowledge, this is a pioneer study investigating the action of digestive enzymes directly in the food matrix (i.e., peanut skin). It is well known that consuming an extract enriched in bioactive compounds is much different from consuming actual food. Therefore, this contribution fills an important gap considering the most common way one would consume this phenolic-enriched part of peanuts.

Dysfunctional oxidative stress processes have been associated with type 2 diabetes, obesity, cardiovascular diseases, and cancer, while several pieces of evidence suggest that adherence to the Mediterranean diet may be beneficial in their prevention and/or management. Reduction in oxidative stress is a crucial mechanism perceived in subjects that present a high adherence to the Mediterranean diet [17]. Phenolics have been found to be the main antioxidants of the Mediterranean diet, being much more consumed than vitamin E (up to 233 times more), vitamin C (up to 28 times more), and carotenoids (up to 192 times more) [18]. These food bioactives also exhibit inhibitory effects toward enzyme metabolic syndrome [19], but it is important to have in vitro evidence that could justify further animal and/or clinical studies in the procurement of novel anti-obesogenic, anti-hyperglycemic, and antiproliferative natural products.

In this scenario, the use of an in vitro method that mimics the human digestive process on peanut skin, which has not been employed in this food matrix before, can result in a scientific advance regarding new strategies to use this by-product, improve production and introduce new ingredients by addressing the release of phenolic compounds and their potential bioactivity. Thus, this study aimed to determine the effects of gastrointestinal and colonic in vitro digestion on phenolics and other chemical constituents, antioxidant activity, the inhibitory effects against α-glucosidase and lipase enzymes, and cytotoxicity of peanut skin.

## 2. Materials and Methods

### 2.1. Reagents and Materials

Type-B proanthocyanidin, 4-methylumbelliferyl oleate, 2,2-diphenyl-1-picrylhydrazyl, 6-hydroxy-2,5,7,8-tetramethylchroman-2-carboxylic acid, 2,2′-azobis(2-amidinopropane) dihydrochloride, 2,4,6-tris(2-pyridyl)-s-triazine; α-amylase, pepsin, type II pancreatic lipase, bile salts, viscozyme, α-glycosidase, and p-nitrophenyl-a-D-glucopyranoside were purchased from Merck (Darmstadt, Germany). Gallic, p-coumaric, quinic, ferulic caffeic, protocatechuic, and ellagic acids, vanillin, quercetin, catechin, epicatechin, epigallocatechin gallate, penicillin, streptomycin, amphotericin B, and RPMI 1640 medium were purchased from Sigma-Aldrich (St. Louis, MO, USA). Formic acid and acetonitrile were purchased from Fisher (Waltham, MA, USA). Cytotox 96 kit was obtained from Promega (Madison, WI, USA), and HCT116 cells (human colorectal carcinoma) were donated by Dr. Bryan Strauss (University of São Paulo School of Medicine, São Paulo, Brazil). Peanut skins were provided by CAP Agroindustry (Dumont, São Paulo, Brazil).

### 2.2. Sample Preparation

Dry-blanched peanut skins were obtained as a by-product of peanut processing (cv. Runner) and kindly donated by CAP—Agroindustrial company (São Paulo, Brazil). Peanut skins were mill-powdered (Mixer Mill MM-400; Retsch, Germany). Ten-millimeter milling vessels were used at 20 Hz for 1 min, sieved through a 100 Mesh sieve, until it reached a granulometry of 150 µm. The resulting powder was portioned into 50 mL vials and stored at −20 °C. The aqueous extract was obtained by mixing 1 g of powdered sample with 20 mL of deionized water. The mixture was homogenized using a dispersing device (Ultra-Turrax) at 14,000 rpm for 3 min, followed by centrifugation at 19,000× *g* for 15 min at 20 °C. The supernatant was recovered and filtered through a qualitative paper filter.

This extraction process was repeated for a total of three cycles. The final volume was adjusted to 60 mL with deionized water, and the peanut skin aqueous extract was centrifuged. The soluble portion of the extract was separated from the insoluble fiber by filtration, and the resulting content (16.7 mg/mL) was stored at −80 °C until use. All analyses were performed under light shielding to minimize polyphenol degradation.

### 2.3. In Vitro Digestion

Simulated digestive fluids were utilized in two phases: phase 1 comprised oral (salivary fluid), gastric (gastric fluid), and upper intestinal (duodenal and biliary fluids) digestion [20]; and phase 2 comprised lower intestinal (colonic fluid) digestion [21]. The full composition of each digestive fluid is shown in Table S1 [20]. Powdered peanut skin (1 g) was mixed with 2 mL of salivary fluid containing α-amylase (pH 6.8 ± 0.2) and incubated for 5 min. Next, 5 mL of gastric fluid containing 2.5 g of pepsin (pH 1.3 ± 0.2) was added to the mixture and incubated for 2 h. The reaction was stopped at −80 °C for 10 min. Then, 5 mL of duodenal fluid containing pancreatin and lipase (pH 8.1 ± 0.2) and 2.5 mL of biliary fluid containing biliary salts (pH 8.2 ± 0.2) were added, and the mixture was incubated for another 2 h. The reaction was again interrupted as described above, followed by centrifugation at 4000× *g* for 10 min and supernatant recovery (phase 1-extract–P1). The final concentration of the soluble extract was 68.9 mg/mL, from which the test dilutions were prepared. The residue was used in phase 2, where 10 mL of colonic fluid containing viscozyme (pH 4.0 ± 0.2) was added and incubated for 12 h. The mixture was centrifuged for 10 min at 4000× *g,* and the supernatant was collected (phase 2-extract-P2) with a final concentration of 96.0 mg/mL. Both extracts were stored at −80 °C. All incubation periods were conducted in a water bath at 37 °C under constant shaking (120/min).

### 2.4. Quantification of Total Phenolic Content

Aqueous and digested extracts were diluted in deionized water (1:100 *v*:*v*). Briefly, 120 µL of diluted extract (or water for a negative control) and 50 µL of Folin–Ciocalteu reagent solution (1:5 *v*:*v*) were added to a 96-well microplate and incubated for 3 min at 25 °C [22]. Next, 30 µL of sodium carbonate solution (200 g/L) was added, followed by incubation at 37 °C for 1 h. The absorbance was measured at 765 nm in a plate reader (SpectraMax M5, Sunnyvale, CA, USA). The quantification of phenolics was determined based on a gallic acid standard curve. Data are described as mg of gallic acid equivalents (GAE) per g of sample.

### 2.5. Determination of Phenolics and Other Chemical Compounds

Thirteen compounds from peanut skin extracts were quantified by high-performance liquid chromatography with diode-array detection (HPLC-DAD); LC 20AD, Shimadzu, Tokyo, Japan) and readings from 190 nm to 400 nm. The extracts were diluted in formic acid 0.1% solution and filtered (0.4-μm mesh size filter). Mobile phases comprised (A) 0.1% formic acid solution and (B) acetonitrile with 0.1% formic acid (by volume). The system was conditioned with 12% B for 10 min, and the run was programmed with the following gradient: 12% B, 0–10 min; 15% B, 11–30 min; 20% B, 31–40 min; 35% B, 41–50 min; and 12% B, 51–65 min. The flux was maintained at 800 µL / min. A 20 µL injection volume was used [23]. The precolumn (C18, 4 mm × 3 mm; Phenomenex Inc., Torrance, CA, USA) and column (C18 Shim-Pack, 5 μm; Shimadzu, Tokyo, Japan) were maintained at 30 °C. The compounds were tentatively identified by comparing retention time and absorbance peak against analytical standards. Quinic acid was detected at 190 nm; protocatechuic acid at 259 nm; ellagic acid at 294 nm; p-coumaric, caffeic, and ferulic acids at 320 nm; type-B proanthocyanidins and gallic acid at 270 nm; catechin, EGCG, and vanillin at 279 nm; and quercetin at 370 nm. Standard curves were constructed by serial dilution of standard solutions and used to quantify individual compounds. Additionally, UPLC-MS/MS identification of free, esterified, and insoluble-bound phenolics was detected in an ABSciex triple Quad 4500 mass spectrometer combined with an Eksigent Ekspert Ultra LC100 and an LC100-XL autosampler system (ABSciex, Concord, ON, Canada). Electrospray was used in negative mode. Chromatographic separation was carried out as published elsewhere [24].

### 2.6. Determination of Antioxidant Properties

The antioxidant activity of the extracts was determined using three different assays. For the DPPH assay [25], P1 and P2 extracts were conveniently diluted in methanol. Then, 20 µL of diluted extract and 180 µL of DPPH methanolic solution were added to transparent microplates. Trolox and methanol were used as positive and negative controls, respectively. After a 30-min incubation period, the absorbance was measured at 515 nm in a plate reader (SpectraMax M5). The percentage of DPPH scavenging was considered according to the following:

[(Absorbance control − Absorbance sample)/Absorbance of control] × 100

Non-linear regression was used to determine the half-maximal scavenging concentration (IC_50_). Data are described as μmol of Trolox equivalents (TE) per g of sample.

For ORAC assay [26], P1 and P2 extracts were diluted in phosphate buffer (75 mM; pH 7.5). Next, 50 µL of diluted extract and 150 µL of fluorescein (93.5 mM) were dispensed in opaque microplates (Fluotrac, Greiner Bio, Sao Paulo, Brazil), followed by incubation at 37 °C for 15 min. Subsequently, 50 µL of an AAPH solution (221 mM) was pipetted into the mixture, and fluorescence was monitored (λ excitation = 493 nm; λ emission = 515 nm; T = 37 °C) at one-minute intervals until the intensity of the signal dropped to 5% or less of the initial value. Trolox and phosphate buffer served as positive and negative controls, respectively. The results were calculated according to the following:

[6.25 × DF × (AUC sample − AUC blank)]/(AUC control − AUC blank),

where DF is the sample’s dilution factor, AUC is the area under the curve, blank refers to the negative control, and control refers to the positive control. Data are described as μmol TE/g of the sample.

For FRAP assay [27], P1 and P2 extracts were conveniently diluted in a 50% methanol solution. Then, 35 µL of the diluted extract and 265 µL of FRAP reagent (containing TPTZ and ferric chloride) were added to a transparent microplate, control (35 µL diluted extract + 265 µL water), and blank (300 µL of 50% methanol). The absorbance was read at 595 nm in a plate reader (SpectraMax M5). Gallic acid was used as a standard. Data are expressed as mg GAE/g of the sample.

### 2.7. In Vitro Pancreatic Lipase Inhibitory Activity

The activity of pancreatic lipase was determined by the production of 4-methylumbelliferone from 4-MUO [28]. Digested extracts were conveniently diluted for this assay. Tris-HCl buffer (pH 8 ± 0.2) was used throughout the experiment. Briefly, 25 µL of diluted extract, 50 µL of a 4-MUO solution (0.1 mM), and 25 µL of a lipase solution (50 U/mL) were added to opaque microplates, followed by incubation at 37 °C for 30 min. Next, 100 µL of sodium citrate solution (0.1 M) was added to the mixture, and fluorescence was measured at 355 nm (excitation) and 460 nm (emission). Negative control (25 µL of water, 50 µL of 4-MUO solution, 125 µL Tris-HCL buffer, 100 µL citrate solution) and blank (all reagents except lipase and citrate solution) were used in this analysis. The percentage of pancreatic lipase inhibition was calculated as follows:

[(Absorbance of control − Absorbance of sample)/Absorbance of control] × 100

Non-linear regression was used to determine the IC_50_. The results are expressed in μg of sample/mL.

### 2.8. In Vitro α-Glucosidase Inhibitory Activity

The activity of alpha-glucosidase was determined by the production of p-nitrophenol from pNPG [29,30]. Digested extracts were conveniently diluted for this assay. Sodium phosphate buffer solution (100 mM; pH 7) was used throughout the experiment. Briefly, 50 µL of diluted extract and 100 µL of an α-glucosidase solution (1 U/mL; pH 7) were added to transparent microplates, followed by incubation at 37 °C for 10 min. Next, 50 µL of pNPG solution (5 mM; pH 7) was pipetted, and the mixture was incubated for another 5 min. Lastly, 100 µL of a calcium carbonate solution (0.1 M) was added, and the absorbance was read at 405 nm in a plate reader (SpectraMax M5). The percentage of α-glucosidase inhibition was calculated according to the following:

[1 − (Absorbance of sample − Absorbance of blank control)/(Absorbance of enzyme control − Absorbance of control] × 100,

where sample refers to the complete reactive media; blank control corresponds to the media minus the enzyme; enzyme control refers to the reactive media minus the sample, and control refers to the reactive media minus both the enzyme and the sample. Non-linear regression was used to determine the IC_50_. The results are expressed in μg of sample/mL.

### 2.9. Cell Viability Assay

The HCT116 cell line was cultured in filtered and sterile RPMI 1640 media supplemented with 2 mM of l-glutamine, 10% fetal bovine serum, 10^5^ units of penicillin, 10 mg of streptomycin, and 25 μg of amphotericin B. Cytotoxicity was evaluated by measuring lactate dehydrogenase levels using the Cytotox 96 kit [31]. Prior to analysis, digested extracts were lyophilized using a bench-top freeze dryer (FDB5503-3L; Operon, Gyeonggi-do, Republic of Korea) and resuspended in cell culture medium at different concentrations (P1 extract: 1–10 mg/mL; P2 extract: 10–25 mg/mL). For the analysis, 50 µL of HCT116 cell suspension (3 × 10^3^ cells) was added to the cell culture, followed by incubation at 37 °C and 5% CO_2_. After 24 h, 50 µL of resuspended extract was added to the medium, and the cells were incubated under the same conditions for 24, 48, and 72 h. The supernatant was collected at selected time points and added to a microplate with 50 µL of Cytotox 96 reagent. After incubation at 25 °C for 30 min, the reaction was stopped by adding 50 µL of a stopper solution. The absorbance of the mixture was measured at 490 nm. Percent cytotoxicity was determined according to the following:

[(Absorbance of sample − Absorbance of negative control)/(Absorbance of positive control − Absorbance of negative control)] × 100

Non-linear regression was used to determine the IC_50_. Data are described as mg/mL.

### 2.10. Statistical Analysis

All assays were conducted in triplicate. Data were analyzed by analysis of variance, Tukey’s posthoc test, or *t*-test, with a significance level of 0.05. Statistical tests were carried out in SPSS 23.0 for Windows 10 Pro (IBM, Armonk, NY, USA).

## 3. Results and Discussion

Overall, most studies evaluating the bioavailability and functional activity of food components utilize aquo–organic extracts to isolate compounds of interest, with the use of different organic solvents, such as alcohols, acetone, ether, and others, to obtain high extraction yields and biological activity. Yet, especially for human consumption applications, these extracts are not adequate to approximate what happens when food is ingested. This study utilized a simulated digestion process to elucidate the difference in phenolics released from peanut skin in each digestion phase.

### 3.1. Total Phenolic Content of Peanut Skin Extracts

The resulting non-digested and digested extracts were analyzed to identify their phenolic composition and compared to the previous literature [32,33]. P1 is related to soluble phytochemicals potentially available in the first intestinal portion, and P2 is related to compounds exposed to the intestinal microbiome and potentially present in the colon.

Table 1 compares the total phenolic contents of aqueous, phase-1, and phase-2 extracts. The simulated digestion process P1 presented approximately 33%, whereas P2 presented 10% of total phenolics compared to the aqueous extract. In other words, phase 1 digestion presented a 2.5-fold higher concentration of phenolic compounds compared with that found in phase 2 digestion (*p* > 0.05). These results show that the in vitro digestion process, simulating food consumption conditions, can release bioactive compounds from the food matrix, making them available up to the absorptive stage.

The concentration of bioactive compounds in food extracts varies according to processing, genetic variability, method, and polarity of the solvents used for the extraction process, with better extraction yields using water and water–alcohol or acetone solutions. Combining physical processes and ethanol solutions, researchers obtained a maximum extraction of total phenolics around 120 mg GAE/g peanut skin [23,34]. When using in vitro digestion directly on the peanut skin powder sample, in a process analogous to human digestion, we obtained a total of around 22.7 mg GAE/g of the sample (P1 + P2). Aqueous extraction presented 68.5 mg GAE/g peanut skin, meaning that about one-third of phenolic compounds were released after the digestive process. Data suggest that the use of digestive methodology produces more realistic results for potential peanut skin phenolic bioavailability.

It is worth noting the importance of analyzing bioactive compounds after digestion for bioavailability studies since the release, degradation, and absorption of bioactive compounds may be affected by different characteristics of food matrices, extraction methods, and other factors. We also highlight that peanut skin presents a great quantity of total phenolic content even after in vitro digestion. Researchers found a total phenolic content of 109.46 mg GAE/g for filtered, freeze-dried, and reconstituted ethanolic extracts [35,36].

### 3.2. Phenolic Compounds, Carboxylic Acids, and Other Organic Compounds from Peanut Skin Extracts

In a previous review, Toomer et al. [6] emphasized that peanuts are a good food source of biologically active polyphenols and that their skin is the main source of these compounds, predominantly catechins and procyanidins. In general, peanut skin extracts have a structure predominantly composed of monomeric and condensed flavonoids, especially proanthocyanidins, and lower concentrations of phenolic acids and stilbene derivatives [37]. The composition of the phenolic and quinic acid profiles found in our aqueous samples corroborates with the findings of other authors [38,39].

Quinic acid, as well as phenolic acids, vanillin (which is a phenolic aldehyde), and some monomeric and dimeric flavonoids (catechin, epicatechin, epigallocatechin gallate–EGCG, quercetin, and type-B proanthocyanidin) were the main compounds positively identified and quantified in our samples (Table 2).

In contrast, quinic acid represented 93% and 85% of all bioactive compounds quantified in P1 and P2 extracts, respectively. This is markedly different from the aqueous extract, where quinic acid represented only 3% of all compounds. Many acids, including quinic acid, occur in combination with other macromolecules in the food matrix, such as carbohydrates [40]. The enzymatic activity of digestive fluids could break these glycosidic bonds to release such compounds, which is evidenced in the different phenolic profiles of aqueous and digested extracts. In turn, the majority of the compounds identified in the aqueous extract belong to the flavanol family (e.g., epicatechin and EGCG). These results indicate, therefore, that the bioactive compounds that are bioavailable upon digestion may not resemble those acquired with traditional extraction methods.

Clifford et al. [41] have reported that compounds with quinic acid epimers, such as acyclic quinic acids, are part of a broad definition of the diverse group of chlorogenic acids (CGA). In general, the acyclic quinic acids are present in coffee drinks, apples, oregano, mate, and other vegetables and are also called quinic acid, L-quinic acid, or 1L-1(OH), 3,4/5-tetrahydroxycyclohexanecarboxylic acid—names recommended by IUPAC [42]. In in vivo studies, there is a predominance of the use or identification of applicability of the isoforms 3-CQA, 4-CQA, and 5-CQA [43], the same compounds that are found after enzymatic hydrolysis after in vitro digestion process [44]. Moreover, this bioavailable natural polyol showed antioxidant, antiviral, antimicrobial, antivascular, anti-inflammatory, and anticancer properties [45,46,47,48]. As an example, oral cancer cells were treated with QA alone and synergistically with cisplatin. Authors concluded that quinic acid inhibits cell proliferation and promotes apoptosis in oral cancer cells (SCC 4) either alone or combined with cisplatin [49].

Minor compounds were also positively identified in our samples, namely, syringic acid, sinapic acid, resveratrol, taxifolin, rutin, and chrysin. However, due to equipment and/or method limitations, a complete phenolic profile was not addressed in this study. Therefore, the results should be interpreted with caution. Most of the compounds were identified in all extracts, except for resveratrol and chrysin, which were detected only in both P1 and P2 extracts. Enzymatic treatment can convert polyphenols bound to phenolic compounds into the free form [50]. Chrysin may be considered a phenolic metabolite formed upon digestion of peanut skin that may also present anticancer activity. Rong et al. [51] demonstrated that chrysin effectively inhibits tumor progression while boosting anti-tumor immunity in a murine model.

Moreover, the most important analogs of resveratrol are derived from hydroxylation, methoxidation, and halogenation and have antioxidant, pro-apoptotic, antitumoral, anti-inflammatory, and antiangiogenic properties, sometimes more potent than their original compound, showing positive effects on human health, and thus, being a compound of therapeutic action against metabolic, cardiovascular, and cancer diseases [52]. The metabolism of resveratrol occurs by the action of digestive enzymes of the intestinal microbiota, whose metabolites are dihydroresveratrol, 4′-dihydroxy-trans-stilbene, and 3,4′-dihydroxybibenzyl [53]. However, the ingestion of the compound and its derivatives has been presented in in vivo studies, with rapid metabolism after oral ingestion [54]. Strategies, such as the use of resveratrol glycosides, allow the ingestion of higher concentrations of active ingredients that could potentially reach the colon, where they are deglycosylated by the microbiota of the gastrointestinal tract [55].

### 3.3. Antioxidant Activity of Digested Peanut Skin Extracts

The different antioxidant assays provide complementary ways to evaluate the antioxidant activity of a given sample. In sum, they all estimate the capacity of peanut skin extracts to neutralize agents that are harmful to cells and tissues; the ORAC assay utilizes peroxyl radicals, the FRAP assay utilizes ferric ions, whereas the DPPH assay utilizes organic free radicals [56]. Table 3 shows the results found for antioxidant activity regarding the elimination of peroxyl radicals, ferric ions, and free radicals and summarizes the results of antioxidant assays using phase 1 and phase 2 extracts. Overall, the P1 extract had greater antioxidant activity than the P2 extract.

When produced in excess, oxidative agents can damage macromolecules, such as proteins, lipids, and DNA, as well as other cellular components. Dysfunctional oxidative stress processes have been associated with cancer, cardiovascular diseases, and type 2 diabetes. Thus, the ingestion of foods containing antioxidant compounds may protect cells and tissues against the detrimental effects of such agents and help maintain an adequate oxidative balance [57,58]. Our results indicate that bioactive compounds from the peanut skin released in the digestion process exhibit antioxidant activity. That can confer an additional benefit of these compounds in the human diet by consumption of the seed with skin or by-products fortified by the addition of peanut skin, such as butter, cookies, pasta, and others [59,60]. The literature shows that roasting peanut skin may boost its antioxidant activity compared with that of raw skin [5].

Molasses ethanol extract gave ORAC antioxidant value of 260 μmol of Trolox/g of the sample [35], and values of 339.4 and 177.4 μmol TE/g were found for grape seed pomace defatted and defatted grape seed pomace, respectively. The antioxidant fiber-rich extract from defatted grape seed pomace reached values of 1439.4 μmol TE/g. The antioxidant fiber-rich extract (AFE) from defatted grape seed pomace presented values of 382.7 μmol TE/g for DPPH methods and 160.9 and 105.5 μmol TE/g for grape seed pomace and defatted grape seed pomace, respectively [36]. For raw cocoa nibs, FRAP values ranged from 10.2 to 13.7 mg GAE/100 g of the sample [61]. Data shows that digested peanut skin presents great antioxidant activity after in vitro hydrolysis.

### 3.4. Enzymatic Inhibitory Activity of Digested Peanut Skin Extracts

**Figure 1:**
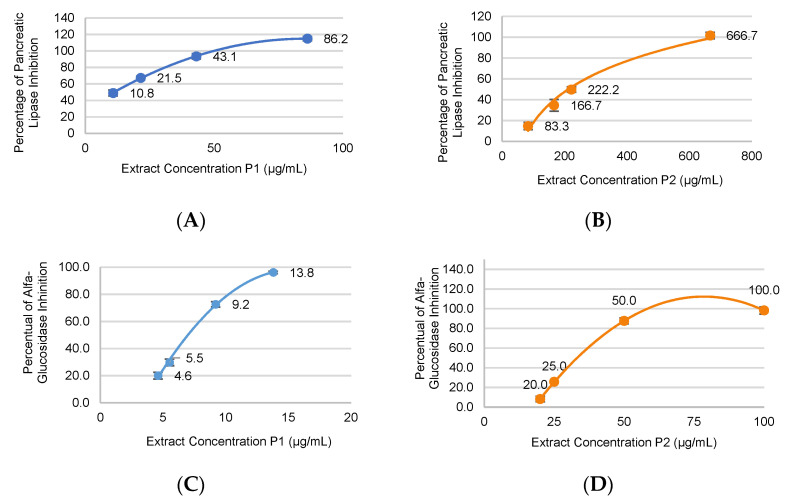
Percentage of inhibition and standard deviation of lipase enzyme (**A**,**B**) and inhibition of α-glucosidase enzyme (**C**,**D**) according to the concentration of digested peanut skin extracts, phase 1 (P1) blue, and phase 2 (P2) orange.

Digested peanut skin extracts displayed inhibitory activity with a positive dose-response relationship. The IC_50_ values for lipase inhibition were 11.2 and 135.6 μg/mL for P1 and P2 extracts, respectively, meaning that the P1 extract was about 10 times more effective than the P2 extract. The IC_50_ values for α-glucosidase inhibition were 7.0 and 33.2 μg/mL for P1 and P2 extracts, respectively. Under this parameter, the P1 extract was about 4 times better than the P2 extract. Overall, the inhibitory activity of the P1 extract was greater than that of the P2 extract, and this was more pronounced for lipase inhibition (Figure 1).

The anthocyanidins present in the black peanut skin extract were tested for their potential to inhibit digestive enzymes, with IC_50_ values of 185.1 μg/mL for pancreatic lipase, 123.4 μg/mL for α-amylase, and 82.8 μg/mL for α-glucosidase [58]. It is noteworthy that the IC50 values for α-glucosidase inhibition found in our study (7.0 and 33.2 μg/mL for P1 and P2 extracts, respectively) are lower than the values found for acarbose (36.0–107 μg/mL) [62], the most studied inhibitor of alpha-glucosidase. In contrast, The IC50 values for lipase inhibition (11.2 and 135.6 μg/mL for P1 and P2 extracts, respectively) were higher than those found for orlistat (0.5–2.7 μg/mL), an anti-obesity drug [63,64].

Nevertheless, our data show greater enzyme inhibition after in vitro digestion, likely due to the structural activity relationships. In fact, type B procyanidin, which is constituted by (epi)catechins as monomeric units, showed an IC50 value of 28.7 μg/mL for α-glucosidase inhibition [65], which is much higher than that of catechin (IC50 = 239.3 μg/mL) [66]. As for the lipase inhibition, epigallocatechin gallate showed a much higher inhibitory effect (22 times higher) than that of epigallocatechin.

One of the strategies to manage hyperglycemia due to dietary carbohydrate absorption is to inhibit enzymes, such as α-glucosidase and thereby reduce carbohydrate hydrolysis and glucose absorption [59]. Mucosal α-glucosidase is well distributed throughout the intestinal mucosa, acting in the breakdown of disaccharides and oligosaccharides [60]. In turn, pancreatic lipase secreted in the early portion of the small intestine is the digestion of dietary fat. This enzyme shows specific activity releasing fatty acids.

With the inhibition of this enzyme, part of the synergistic course of lipid digestion and sequentially its intestinal absorption will be influenced [67]. The modulation of enzymes responsible for the digestion of macronutrients, such as carbohydrates (α-glucosidase) and lipids (pancreatic lipase), represents a unique strategy to treat and prevent metabolic disorders, such as hyperlipidemia, obesity, and hyperglycemia [10]. Partially inactivating these enzymes may result in greater lipid excretion, less lipid absorption, and lower post-prandial blood glucose levels [67]. In this context, the inclusion of foods rich in polyphenols, such as peanut skin, in one’s diet is of great relevance.

In a mice model study, supplementation with polyphenols extracted from peanut skin lessened the effects of a typical Western diet (i.e., rich in saturated fat and cholesterol). Mice that received supplementation displayed less hepatic fat deposits, lower triglyceride levels, lower expression of genes related to lipogenesis, lower body weight, lower plasma glucose levels, smaller liver and spleen sizes, and lower hepatic glycogen and cholesterol levels compared with those that did not receive supplementation [6]. Our pancreatic lipase inhibition data corroborate this finding, which may be related to an anti-lipidemic effect.

Some studies in animal models demonstrated that phenolic compounds may act synergistically with commercial anti-hyperglycemic drugs, thus suggesting that these natural compounds could be used to decrease the dose of these drugs in type 2 diabetes patients [68,69].

In summary, our results from in vitro digestion indicate that peanut skin may contribute to preventing and managing diseases resulting from an imbalance in the metabolism of carbohydrates, such as type 2 diabetes and, to a lower extent, dyslipidemia and obesity, as part of a healthy diet pattern that includes adequate amounts of dietary sources of bioactive compounds. Further studies in animal models or clinical trials are necessary to confirm that peanut skin as an industrial by-product may be used in the composition of functional foods with anti-diabetic and anti-lipidemic properties.

### 3.5. Cell Viability

**Figure 2:**
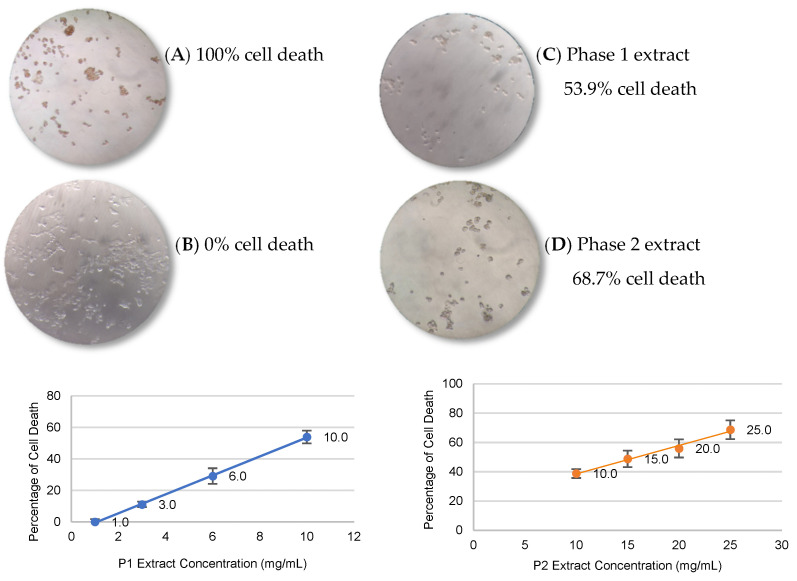
Cell proliferation analysis in HCT116 cells. Positive control (**A**); Negative control (**B**); Digested peanut skin extract Phase 1 (P1) to 10 mg/mL (**C**); and Digested peanut skin extract Phase 2 (P2) to 25 mg/mL (**D**) after 48 h (photos taken by the first author). Plots of administered sample concentration by percentage of cell death for P1 (blue) and P2 (orange).

The IC_50_ values for cytotoxicity were 9.4 and 15.9 mg of digested extract/mL of RPMI medium for P1 and P2 extract, respectively. Figure 2 illustrates the cytotoxic effects of digested peanut skin extracts on HCT116 cell viability. For both extracts, after 48 h, cells undergoing division, as well as engorged and condensed cells, were still visible.

The greatest cytotoxic effects were observed in the incubation time of 48 h, which is in accordance with that reported [70,71]. These authors verified that, for several compounds, incubation times had to surpass 48 h to induce dose-dependent cellular death. Savatović et al. [70] evaluated the antiproliferative activity of Granny Smith pomace extract on the HT-29 cell line, reporting the IC_50_ corresponding to 23 mg/mL.

According to the literature, a substance is considered cytotoxic, poorly cytotoxic, or not toxic when percent cell viability is lower than 50%, between 50% and 70%, and higher than 70%, respectively [71]. Candela et al. [32] verified that ethanolic extracts of peanut skin at 250 mg/kg had no genotoxic effects on normal cells. Similarly, Rossi et al. [72] reported that ethanolic extracts of peanut skin did not exert cytotoxic effects on epithelial cells up to 300 μg/mL.

An individual of average weight (70 kg) who consumes a moderate amount of peanuts (28–42 g/day) ingests <100 mg of peanut skin per kg of body weight [73]. Taken together, this evidence indicates that the intake of peanut skin is safe and does not result in acute cytotoxic effects at the tested doses in normal cells.

Nevertheless, cancer is a highly complex multistep condition that includes inflammation and other chemical or biological stressor agents [74]. Oxidative stress plays a critical role in the pathogenesis of cancer, especially free radicals, in the production of oxidative damage that affects metabolic pathways related to cell proliferation and inflammation. In normal cells, Nfr2 regulates cytoprotective and antioxidant gene expression, eliminating ROS through the upregulation of enzymes responsible for the induction and production of antioxidant molecules [75]. In contrast, overexpression of Nrf2 is linked to tumor growth [76,77]. In cases in which the tumor was already developed, Nrf2 inhibitors can be used as anticancer agents. One of the effective Nrf2 inhibitors is chrysin [78], which was found in our samples. Hence, other compounds with antioxidant properties may also present prooxidant effects in the tumor microenvironment [79].

As previously discussed, the literature shows that quinic acid was able to inhibit cell proliferation and direct oral cancer cells (SCC 4) to apoptosis in isolation and when associated with cisplatin [49]. Another study showed that combined treatment with quinic acid and anti-programmed cell death protein 1 mAb was able to significantly decrease tumor growth and enable longer survival times in mice with bowel cancer, compared to any treatment isolated in cells of colon cancer inflamed by T cells [12]. Furthermore, peanut phenolics seem to affect histone acetylation in MCF-7 and HeLa cells through several studied pathways, resulting in cancer prevention [70]. In parallel, in human hepatic carcinoma cells (HepG2 cells), the biological effects of an ethanolic peanut shell extract showed hyperglycemia-induced hepatic responses with attenuation of ALT activity, suggesting a protective effect against cellular dysfunction in this cell type [80].

Our findings provide important information about chemical compounds and the potential biological activity of peanut skin. This by-product may be beneficial for human health, as indicated here mostly by chemical-based assays after in vitro gastrointestinal digestion. However, it is important to mention that additional cell-based assays, animal models, or, preferentially, human trials are crucial to adequately support the biological potential of peanut skin. Collectively, our data and previous studies suggest that peanut skin could be sustainably used by the food industry.

## 4. Conclusions

In general, the extracts obtained from the in vitro digestion of peanut skin had a diverse phenolic profile and distinct biological potential based on their inhibitory capacity toward α-glucosidase and lipase as well as antioxidant activity. In contrast, they displayed low cytotoxic effects on HCT116 cells, which are human colorectal carcinoma cells. According to our data and the literature, we found that significant amounts of bioactive compounds are released after the digestion process of peanut skin and that the digested products may act in biological processes of the human metabolism, potentially exhibiting antioxidant, antidiabetic, anti-lipidemic, and anti-proliferative properties of human colorectal carcinoma cells. Therefore, peanut skin may be incorporated into a healthy diet, included in the formulation of novel foods, and used as a functional ingredient that possesses bioavailable compounds. Despite the evidence regarding the beneficial effects of peanut skin, this layer is commonly removed during food processing and is considered a waste product of the food industry. However, our results show that this by-product has the potential for human health applications and can be used as a rich source of antioxidants.

## Figures and Tables

**Table 1:** Total phenolic contents of peanut skin aqueous and digested extracts.

Extract	Total Phenolic Content (mg GAE/g of Sample)
Aqueous	68.5 ± 0.6 ^a^
P1 extract	16.1 ± 0.3 ^b^
P2 extract	6.6 ± 0.1 ^c^

Results are expressed as mean ± standard deviation. Aqueous (Aw): undigested extract. Phase 1 (P1): soluble extract from oral, gastric, and duodenal digestion of peanut skin. Phase 2 (P2): insoluble residue from P1 + simulated colonic digestion of peanut skin. Values followed by different letters (a–c) differ statistically at *p* < 0.05 (Tukey test) for different peanut skin extracts.

**Table 2:** Phenolics and quinic acid of peanut skin quantified in aqueous and digested extracts.

Compound	Concentration (µg/g of Sample)		
	Aqueous Extract	P1 Extract	P2 Extract
Type-B proanthocyanidin	0.4 ± 0.0 ^a^	20.2 ± 0.80 ^c^	2.5 ± 0.1 ^b^
Quinic acid	123.5 ± 6.2 ^a^	25,459.9 ± 512.50 ^c^	6667.1 ± 102.4 ^b^
Gallic acid	50.01 ± 0.9 ^b^	66.7 ± 0.80 ^c^	12.8 ± 0.1 ^a^
Protocatechuic acid	17.0 ± 2.3 ^a^	231.0 ± 13.30 ^c^	72.2 ± 0.8 ^b^
Catechin	325.5 ± 1.2 ^b^	363.6 ± 16.6 ^c^	270.0 ± 6.3 ^a^
Caffeic acid	135.6 ± 2.1 ^b^	342.4 ± 5.9 ^c^	76.5 ± 0.1 ^a^
Epicatechin	672.8 ± 47.7 ^b^	324.3 ± 27.7 ^a^	319.8 ± 2.5 ^a^
Epigallocatechin gallate	1417.4 ± 24.0 ^c^	157.8 ± 1.2 ^a^	185.6 ± 10.2 ^b^
Vanillin	259.5 ± 21.8 ^b^	37.5 ± 3.8 ^a^	16.4 ± 1.2 ^a^
Coumaric acid	1.2 ± 0.3 ^a^	33.0 ± 0.5 ^c^	14.0 ± 2.3 ^b^
Ferulic acid	77.8 ± 1.8 ^b^	7.2 ± 0.2 ^a^	6.2 ± 0.1 ^a^
Ellagic acid acid	720.7 ± 11.1 ^c^	190.5 ± 5.6 ^b^	99.0 ± 1.90
Quercetin	2.7 ± 0.0 ^c^	0.8 ± 0.0 ^b^	nd ^a^

Results are expressed as mean ± standard deviation. Aqueous (Aw): undigested extract. Phase 1 (P1): oral, gastric, and duodenal digestion of peanut skin. Phase 2 (P2): simulated colonic digestion of peanut skin; nd: not detected. Values within lines followed by the same superscript lowercase letter (a–c) do not differ statistically at *p* < 0.05 (Tukey test) for different peanut skin extracts.

**Table 3:** Antioxidant activity and reducing power of digested peanut skin extracts.

Extract	ORAC *	FRAP *	DPPH *
	(µmol TE/g of Sample)	(mg GAE/g of Sample)	(µmol TE/g of Sample)
Phase 1	129.0 ± 18.8 ^a^	7.0 ± 0.3 ^a^	10.9 ± 0.0 ^a^
Phase 2	53.5 ± 3.7 ^b^	2.1 ± 0.1 ^b^	5.7 ± 0.0 ^b^

* ORAC, FRAP, and DPPH values are expressed as mean ± standard deviation. Phase 1 (P1): oral, gastric, and duodenal digestion of peanut skin. Phase 2 (P2): simulated colonic digestion of peanut skin. Values within the column followed by different superscript lowercase letters (a–b) differ statistically at *p* < 0.05 (Tukey test) for different peanut skin extracts.

## Data Availability

The data presented in this study are available on request from the first author and A.C.d.C.

## Supplementary Materials

The following supporting information can be downloaded at: https://www.mdpi.com/article/10.3390/antiox12071356/s1.
