# Supplementary material for: In Vitro Digestion of Peanut Skin Releases Bioactive Compounds and Increases Cancer Cell Toxicity
Source: Antioxidants (Basel). 2023 Jun 28;12(7):1356. doi: 10.3390/antiox12071356 (PMC10376574; doi:10.3390/antiox12071356)
Supplement: Supplementary file 1 [file antioxidants-12-01356-s001.zip › antioxidants-2440197-supplementary.pdf]

Supplementary Material

Supplementary Table S1. Composition of the digestive fluids utilized for the simulated digestion assay

Compound	Digestive fluid				
	**Salivary**	**Gastric**	**Duodenal**	**Biliary**	**Colonic**
Ultrapure water	500 mL	500 mL	500 mL	500 mL	500mL
NaCl	58.50 mg	2.75 g	7.01 g	5.27 g	-
KCl	74.50 mg	0.82 g	0.56 g	0.38 g	-
$NaHCO_3$	1.05 g	-	3.39 g	5.79 g	-
$CaCl_2 \cdot 2H_2O$	-	0.39 g	-	-	-
NaH_2PO_4	-	0.27 g	-	-	-
KH_2PO_4	-	-	800 mg	-	6.8 g
NH_4Cl	-	0.30 g	-	-	-
$MgCl_2$. $6H_2O$	-	-	106.76 mg	-	-
Urea	0.20 g	0.08 g	0.1 g	0.26 g	-
Concentrated HCl	-	6.50 mL	0.18 mL	0.15 mL	-
Enzyme*	α-amylase, 1.0 g	Pepsin, 2.5 g	Pancreatin, 9.0 g; lipase, 1.5 g	Biliary salts, 12.0 g	Viscozyme, 10 mg
pH	6.8 ± 0.2	1.30 ± 0.02	8.1 ± 0.2	8.2 ± 0.2	4.0 ± 0.2

*Enzymes were added to the digestive fluid at the time of experiment
